# Patient Needs and Lived Experiences Inside the Multiplace Hyperbaric Chamber: Insights from a Phenomenological Study

**DOI:** 10.3390/nursrep16020054

**Published:** 2026-02-05

**Authors:** Dalmau Vila-Vidal, Angel Romero-Collado, David Ballester-Ferrando, José M. Inoriza, Carolina Rascón-Hernán

**Affiliations:** 1 Hospital de Palamós (Fundació Hospital de Palamós—Serveis de Salut Integrats del Baix Empordà), Carrer Hospital, 17-19, 17230 Palamós, Spain; dvila@ssibe.cat (D.V.-V.);; 2 Nursing Department, Nursing Faculty of Girona University, Carrer Emili Grahit, 77, 17003 Girona, Spain; david.ballester@udg.edu (D.B.-F.);

**Keywords:** health communication, hyperbaric oxygenation, nursing care, qualitative research

## Abstract

**Background/Objectives**: Hyperbaric Oxygen Therapy (HBOT) involves breathing oxygen at pressures greater than atmospheric levels and is used to treat diverse clinical conditions. However, little is known about the lived experiences and perceived needs of patients undergoing scheduled treatment in multiplace hyperbaric chambers, where nurses play a key role in support, safety, and communication. This study aimed to explore the perceptions, expectations, and needs of patients receiving scheduled HBOT sessions in a multiplace chamber in a hospital setting. **Methods**: A qualitative phenomenological design was used. Participants were recruited consecutively among adults who had completed at least 10 HBOT sessions and demonstrated adequate cognitive function. Individual semi-structured interviews were conducted between January and March 2023 in locations chosen by participants. Interviews were audio-recorded, transcribed, and validated by participants. **Results**: Twelve participants (eight men, four women; aged 25–84 years) were included. Four thematic areas emerged: (1) Biopsychosocial lived experiences, including initial uncertainty, physical discomfort such as ear pressure or mask-related issues, and progressive recognition of therapeutic benefits. (2) Interpersonal relationships, highlighting trust, security, and emotional support provided mainly by nurses. (3) Communication experiences, with participants expressing satisfaction but requesting clearer, earlier information on procedures, risks, and expected sensations. (4) Structural and organizational factors, where transportation logistics and treatment scheduling were significant sources of fatigue and discomfort. **Conclusions**: Patients valued HBOT and perceived notable health improvements, while identifying specific unmet informational and organizational needs. These findings suggest the importance of nurse-led educational interventions to enhance preparation, reduce anxiety, and optimize patient experience during HBOT.

## 1. Introduction

Hyperbaric oxygen therapy (HBOT) consists of breathing oxygen at a pressure higher than the local atmospheric pressure, and is applied for the prevention or treatment of a wide range of acute and chronic conditions. The term HBOT is restricted to treatments in which the partial pressure of oxygen exceeds 1.5 atmospheres absolute (ATA) for at least 60 min, excluding compression and decompression phases [1].

From a clinical standpoint, HBOT is indicated for conditions such as carbon monoxide poisoning, retinal artery occlusion, radiation-induced tissue damage, and diabetic foot ulcers, among others [2,3].

Although widely used, HBOT remains unfamiliar to many patients and is sometimes mistakenly only associated with diving medicine [4].

HBOT in hospital settings is frequently delivered in multiplace chambers [1], enclosed environments with seating capacity for several individuals receiving simultaneous treatment. The number of sessions varies considerably depending on the underlying condition, ranging from a few sessions for acute indications [5] to more than 70 for chronic or complex cases [6]. HBOT is a safe procedure with a low prevalence of adverse effects [7,8], although these may begin to appear after approximately ten hyperbaric sessions [9].

This intensive and highly technological setting can elicit emotional and physical responses, including uncertainty, anxiety, discomfort related to pressure compensation, and the impression of entering an unfamiliar environment [4,10]. In addition to these reactions, patients have also reported ear discomfort, transient ocular effects [9], noise and fluctuations in temperature within the chamber, as well as discomfort associated with the mask or hood and the monotony of long sessions [11]. Previous qualitative studies have described patients’ feelings of approaching “an unknown world” [12] or “walking into the unknown” [10], highlighting the psychological impact of undergoing treatment in a confined space over repeated sessions.

Nurses play a central role in HBOT delivery, as they provide continuous monitoring, guidance on pressure compensation techniques, reassurance, and support during the session [13]. Understanding patients’ experiences is essential for informing patient-centred nursing interventions, improving communication practices, and enhancing the safety and comfort of the treatment environment [14]. Identifying patients’ unmet needs may also contribute to the development of tailored educational programmes and improvements in organizational processes.

Despite the increasing use of HBOT, qualitative evidence describing patients lived experiences and perceived needs in hospital-based multiplace chambers remains limited, particularly regarding informational needs, comfort-related challenges, and organizational factors that influence treatment burden. This knowledge gap is clinically relevant for nursing practice, as nurses provide continuous monitoring and support inside the chamber and play a central role in patient education and reassurance. Therefore, exploring patients’ perspectives is necessary to inform patient-centred nursing interventions and service improvements in hyperbaric units.

The aim of this study was to explore the perceptions and needs of individuals undergoing scheduled treatment sessions in a hospital-based multiplace hyperbaric chamber.

## 2. Material and Methods

### 2.1. Design

This study employed a qualitative phenomenological design to explore in depth how patients perceive and make sense of scheduled HBOT sessions in a multiplace chamber, with a focus on their lived experiences and perceived needs. A phenomenological approach was considered appropriate as it enables exploration of the meanings and subjective dimensions of treatment experiences that are not readily captured through quantitative measures. The study followed the ‘Consolidated criteria for reporting qualitative research’ (COREQ) [15].

### 2.2. Study Setting and Recruitment

Participants were recruited using consecutive non-probabilistic sampling among eligible patients. Individuals who had been prescribed hyperbaric oxygen therapy (HBOT) within the three months preceding the start of the study were invited to participate. Recruitment took place during the initial clinical interview routinely conducted by members of the hyperbaric chamber team. Eligible patients were approached, provided with verbal and written information about the study, and invited to participate. Enrolment continued progressively until data saturation was reached, defined as the point at which no new relevant information emerged from the interviews. All eligible patients who were invited to participate agreed to take part in the study, and no refusals were recorded.

Before beginning HBOT, all patients attended a clinical consultation with a hyperbaric physician who explained the treatment procedure, its technical characteristics, and potential risks, and addressed any questions raised by the patients. Written informed consent was obtained during this consultation.

HBOT was delivered inside a HAUX-STARMED 2200/5.5/KB (Haux, Karlsbad-Ittersbach, Germany) multiplace hyperbaric chamber with a maximum capacity of eight people. Each treatment session lasted 125 min at a pressure of 2.4 bar (240 kPa). Oxygen was administered in three 30 min periods, interspersed with 5 min air breaks. An experienced nurse remained inside the chamber throughout all HBOT sessions to monitor patients and provide support as needed.

### 2.3. Inclusion and/or Exclusion Criteria

Inclusion criteria were: adults aged 18 years or older; absence of cognitive impairment, assessed using the Pfeiffer Short Portable Mental Status Questionnaire with a score ≤ 2 [16]; current treatment in the hyperbaric chamber; and completion of at least ten HBOT sessions.

Cognitive screening using the Pfeiffer Short Portable Mental Status Questionnaire was applied as a precautionary measure to ensure participants’ ability to engage in in-depth qualitative interviews. No patients were excluded based on cognitive impairment, as all invited participants obtained a Pfeiffer score ≤ 2.

The criterion of having completed at least 10 HBOT sessions was established to ensure sufficient exposure to the chamber environment and treatment routine. This threshold was informed by previous evidence indicating that certain treatment-related discomforts and adverse effects of hyperbaric oxygen therapy tend to emerge after repeated sessions, often becoming apparent after approximately ten sessions [9]. By this stage, participants are also able to reflect on both initial and ongoing experiences, including adaptation processes and evolving care needs.

### 2.4. Data Collection

Data were collected between January and March 2023 through individual semi-structured interviews conducted either in an office within the hyperbaric medicine unit, at a primary healthcare centre, or in participants’ homes, according to their preference. Seven participants chose to be interviewed in their homes. For these participants, interviews were conducted within ten days after their most recent HBOT session, ensuring that treatment-related experiences were recent while allowing flexibility to accommodate participants’ preferences and availability. Each interview lasted between 45 and 60 min, depending on the participant’s availability and depth of discussion. During the interviews, the researcher took field notes in a dedicated notebook to document relevant aspects of non-verbal communication, such as pauses, emotional expressions, visible discomfort, or changes in tone. These observations were used to contextualize participants’ verbal accounts and to support interpretative depth during analysis, rather than as independent coding units. The interview guide is presented in Table 1.

The interview guide was developed based on a review of the literature on patient experiences of HBOT and on the clinical expertise of the research team. Questions were designed to elicit detailed narratives regarding informational needs, physical sensations, comfort, and organizational aspects of care. The guide was reviewed within the research team for clarity and relevance and was applied flexibly, allowing prompts and follow-up questions to explore issues raised by participants.

All interviews were audio-recorded and transcribed verbatim, and the transcripts were complemented with field notes taken during the sessions.

Before proceeding with the analysis, transcripts were returned to participants to allow them to review, clarify, or amend any information (member checking). None of the participants requested modifications.

### 2.5. Data Analysis

Data analysis followed Braun and Clarke’s thematic analysis approach [17]. The first author, a nurse researcher with training in qualitative methods, conducted all interviews and led the initial analytic process. To ensure deep familiarization with the data, the researcher listened to the audio recordings, read the transcripts repeatedly, and integrated field notes documenting relevant non-verbal communication.

An inductive coding process was applied. No pre-defined codes, keywords, or categories were established prior to analysis. Transcripts were imported into ATLAS.ti (version 23) to support systematic data management. Initial coding was conducted line by line, remaining close to participants’ own words and meanings. Codes were generated directly from the data and reflected participants’ experiences, perceptions, and interpretations of hyperbaric oxygen therapy.

Field notes documenting non-verbal communication—such as pauses, emotional expressions, visible discomfort, or changes in tone—were integrated during the familiarization and interpretation phases to enhance contextual understanding of the verbal data. These observations were used to support interpretative depth and clarify emotional intensity or situational meaning but did not constitute independent or pre-defined coding units.

Codes were iteratively compared across transcripts and progressively grouped into broader categories based on conceptual similarity, shared meaning, and recurring patterns across participants. This process involved constant comparison between codes, categories, and the full data set, allowing for refinement of category boundaries and consolidation of analytically coherent groupings.

A second researcher, also experienced in qualitative analysis, independently reviewed the coded transcripts within ATLAS.ti to enhance analytical rigour. Regular peer debriefing sessions were held among members of the research team to discuss coding decisions, explore alternative interpretations, and ensure consistency in category development. Any discrepancies were resolved through discussion until consensus was achieved.

Themes were developed iteratively by examining relationships among categories and refining their scope, coherence, and representativeness. Theme review was conducted through a researcher-led, iterative process rather than by the software itself. Preliminary themes were systematically compared against the complete data set to ensure internal coherence, consistency across participants, and accurate representation of participants’ accounts.

An audit trail was maintained within ATLAS.ti by preserving successive versions of the coding framework, documenting code definitions and changes, recording analytic memos, and retaining explicit links between raw data excerpts, codes, categories, and themes. These records ensured transparency, traceability, and dependability of analytical decisions throughout the coding and theme development process.

The use of ATLAS.ti supported transparency, traceability, and organization throughout the analytical process, consistent with COREQ [15] recommendations.

### 2.6. Ethical Considerations

All participants were informed about the purpose of the study, and those who agreed to take part signed a written informed consent form. Data were pseudo-anonymized using an identification code accessible only to the principal investigator. Participants were informed that they could withdraw from the study at any time and without providing any justification. The study protocol received approval from Hospital de Palamós Research Committee (protocol code project 44519) and Ethics Committee of Research with Medicines (CEIm Girona, protocol code project 175_19).

During the interviews, researchers were attentive to signs of emotional discomfort. Participants were explicitly informed that they could pause or discontinue the interview at any time without providing a reason. In the event that emotional distress had arisen, appropriate clinical support pathways within the hospital would have been activated. No interviews required interruption or additional support.

### 2.7. Rigour and Reflexivity

Rigour and reflexivity were ensured following the COREQ criteria [15]. The research team consisted of nurses with clinical expertise in hospital care and specific training in qualitative methods. None of the interviewers were part of the hyperbaric chamber clinical team, which helped minimize potential role-related influence on participants’ responses.

Reflexive discussions were maintained throughout the study as a methodological strategy to identify, critically examine, and make explicit researchers’ assumptions, professional backgrounds, and potential influences on data interpretation. Rather than generating consensus-driven interpretations, these discussions were used to question preliminary analytic decisions and to ensure that interpretations remained grounded in participants’ accounts. Reflexivity was therefore employed to enhance transparency and credibility, acknowledging the interpretative role of researchers while minimizing unexamined bias.

Regarding the relationship with participants, contact was initiated during the routine clinical process when individuals were invited to participate. To reduce perceived power imbalances, researchers clarified their independence from the clinical staff and emphasized that participation would not affect treatment or care.

Methodological rigour was supported through audio-recorded interviews, verbatim transcription, and the integration of field notes documenting non-verbal communication. A systematic and iterative thematic analysis was conducted, with regular peer debriefing among the research team to ensure consistency in coding and category development. Data saturation was achieved after twelve interviews, when no new relevant themes emerged.

Credibility was further enhanced by offering participants the opportunity to review their transcripts (member checking), although no changes were requested. Confirmability was strengthened by maintaining an audit trail of analytic decisions and coding processes within ATLAS.ti. Transferability was supported through a detailed description of the study setting, participant characteristics, and organizational aspects of hyperbaric chamber treatment. Reflexive engagement was maintained throughout the research process to ensure that findings remained closely linked to participants’ accounts.

## 3. Results

The sample consisted of four women and eight men, with a mean age of 76.25 and 71.5 years, respectively. Table 2 presents the characteristics of the study participants. Regarding interview location, seven participants chose to be interviewed in their homes, three opted for their primary healthcare centre, and two preferred the hyperbaric medicine unit.

**Figure 1:**
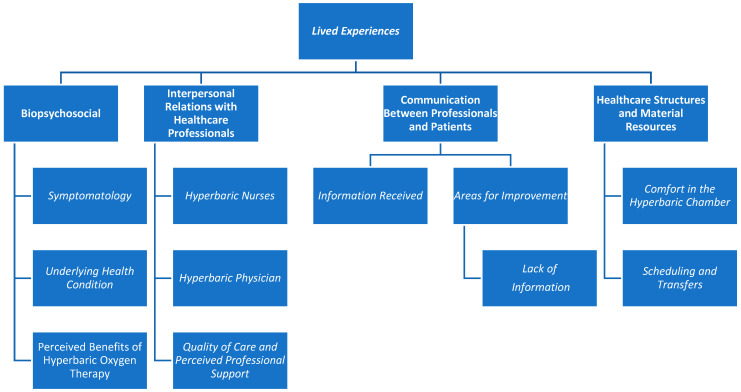
Lived experiences of people treated in the hyperbaric chamber.

Figure 1 displays the four thematic areas identified during the analysis, together with their main and secondary categories. The first thematic area, Biopsychosocial Lived Experiences, comprises three main categories. The second area, Lived Experiences Through Interpersonal Relations with Healthcare Professionals, includes three main categories. The third thematic area, Lived Experiences Related to Communication Between Professionals and Patients, encompasses two main categories and one secondary category. Finally, the fourth area, Lived Experiences Related to Healthcare Structures and Material Resources, is composed of two main categories.

### 3.1. Theme 1: Biopsychosocial Lived Experiences

This thematic area comprises three main categories: symptomatology, underlying health condition, and perceived benefits of hyperbaric oxygen therapy.

#### 3.1.1. Symptomatology

Most participants reported not experiencing anxiety during the hyperbaric oxygen therapy sessions. However, several described a sense of unease or nervousness on the day before treatment or upon entering the chamber for the first time. These feelings were commonly linked to uncertainty and the unfamiliarity of the environment. Participant statements illustrate this variability:

- U2: “…no, I did not feel anxious or experience any strange sensation (…) no distress at all.”
- U8: “I imagined it would be a larger space. It created a bit of… well, of wondering what this is and how it works.”
- U6: “I felt a little nervous at the beginning of the first session…”
- U8: “Rather than anxiety, I felt… curiosity about the unknown.”

These accounts highlight that while overt anxiety was uncommon, anticipatory uncertainty and mild nervousness were characteristic during the initial encounter with the hyperbaric chamber.

Only one participant reported experiencing claustrophobia, which was linked to a previous traumatic event. The remaining participants stated that they did not experience claustrophobic feelings during treatment.

- U1: “I can’t stay in enclosed spaces for too long because I start to feel very nervous…”

Ear pressure compensation was another area in which experiences varied. One participant described significant difficulty during the first session, reporting intense pain and the urge to leave the chamber until they learned how to perform the compensation technique properly. With time and practice, symptoms improved. In contrast, most participants reported only mild, transient discomfort, explaining that they were taught how to compensate from the first day and that any pressure-related symptoms diminished quickly.

- U1: “The ears… that was terrible for me. If I could have left, I would have it…”
- U4: “They taught me how to compensate my ears on the first day, inside the chamber (…) Just a bit of discomfort, some days a little clicking sound, but nothing important.”
- U7: “I had a bit of a hard time the first day, but less on the second, and afterwards my ears didn’t hurt at that moment anymore.”

The need to urinate or defecate during treatment emerged as one of the most significant sources of concern and discomfort for participants. The need to urinate or defecate during treatment emerged as one of the most significant sources of concern and discomfort for participants. Although this issue was more frequently emphasized by older participants, younger participants also reported similar concerns, describing anxiety related to the duration of the session, the confined environment, and the perceived lack of control over bodily needs once treatment had begun. For these participants, discomfort was primarily associated with anticipatory stress and fear of being unable to manage elimination needs while inside the chamber, rather than with age-related physical limitations. All individuals undergoing HBOT sessions described this as a major worry, noting the embarrassment and practical difficulties associated with experiencing elimination needs inside the chamber. To prevent this, participants routinely adopted strategies such as going to the bathroom immediately before entering the session.

- U4: “Before entering the chamber, I would try to go to the toilet, and that was it—I could hold it well.”
- U5: “I went before going in… I was worried about needing to use the bathroom inside the chamber.”
- U9: “It actually happened to me—I had diarrhoea (…). Luckily, I was alone that day. It was a weekend session, and I felt so, so embarrassed… I was really ashamed.”

These accounts highlight that elimination needs represent a key biopsychosocial challenge during HBOT, contributing to anticipatory stress and, in some cases, significant emotional distress.

Participants reported varying levels of information regarding the possibility of visual changes during HBOT. Some indicated that they had not been informed of this potential effect, while others recalled receiving an explanation but did not personally notice any change in vision. In most cases, participants were unable to clearly attribute minor fluctuations in visual acuity to the hyperbaric treatment. One participant, who had suffered a stroke, described a noticeable improvement, whereas another reported transient visual disturbance that was explained by the hyperbaric physician as a temporary effect.

- U4: “They mentioned I might develop some myopia, but I didn’t notice anything.”
- U7: “Honestly, I haven’t noticed much visually. Sometimes I think I might see a bit better, but no—I think it’s more or less the same.”
- U1: “For me it’s been great because I couldn’t drive before; now I can. I see well from far away and up close. Before, I could only see half as well.”
- U6: “I had done maybe thirty-five or forty sessions when the doctor mentioned it… and when I reached sixty or sixty-five, I told the doctor, ‘Look, something is happening—I think I have a vision problem because I can’t see well.’ And he told me, ‘Yes, we know your vision is being affected a little; the cornea dries out a bit. But the benefits outweigh this, and in about three months your vision will recover.’”

These accounts illustrate a spectrum of patient experiences regarding visual changes, ranging from no perceived alteration to notable improvement or temporary discomfort, highlighting the importance of clear anticipatory guidance about potential visual effects of HBOT.

#### 3.1.2. Underlying Health Condition

Participants frequently referred to challenges related to their underlying health conditions, both inside and outside the hyperbaric chamber. These included issues such as bleeding or pre-existing ocular conditions—like cataracts or retinal detachment—that made the time spent inside the chamber more difficult. Many participants expressed that their primary concern was not the hyperbaric treatment itself but rather the severity of their baseline condition, which in several cases involved neoplastic disease. For some, HBOT represented the last available therapeutic option.

Several accounts illustrate the impact of these underlying conditions:

- U2: “…they even had to give me a bit more blood, and at one point the urologists came down to the chamber area to unblock the catheter while I was in the hyperbaric unit. So, the only problem I had during the hyperbaric sessions was because I had the catheter. When I finished the session, they would tell me to wait, and sometimes they would unblock it for me.”
- U2: “It wasn’t a problem with the chamber; it’s my eyesight. In my left eye I have a cataract, and I’ve had a retinal detachment—several problems…”
- U3: “The accident I had was the worst part.”
- U4: “My worry was the bleeding. Now it’s been almost fifteen days without bleeding… it comes in small bouts, you know, but I’m doing better.”
- U5: “My concern was that the wound would close, that the ulcer would heal. I didn’t worry about anything else… what mattered was healing.”
- U7: “I was worried about the radiotherapy, about the colon cancer… that concerned me.”
- U9: “I was in a very delicate moment—my daughter’s father, who was not my partner, had just died, and I was dealing with breast cancer… we were going through a very difficult situation.”

These narratives show that participants’ greatest difficulties and emotional burdens were closely tied to their underlying medical conditions and personal circumstances. HBOT was often perceived as secondary to these broader health challenges, yet also as a potentially crucial opportunity for improvement.

#### 3.1.3. Perceived Benefits of Hyperbaric Oxygen Therapy

For all participants, HBOT provided significant health benefits, particularly because in many cases it represented their last available therapeutic option. Expectations for improvement were high across the sample, and participants consistently reported that these expectations were fulfilled. Overall satisfaction with the treatment outcomes was strong. Participants also highlighted that HBOT is a lengthy process and that improvements generally do not appear immediately but become evident progressively over time.

The following quotations illustrate these perceived benefits:

- U1: “It has worked wonderfully for me because I couldn’t drive before, and now I can… I’m very happy because I fought hard for my vision.”
- U2: “I started because of the bleeding, hoping—really hoping—that it would stop.”
- U6: “They had told me the chamber treatment might help me… I just wanted them to try to solve what was a massive problem… and I understood that the chamber would fix it. My expectations have been more than fulfilled—far beyond what I thought.”
- U8: “…it was absolutely worth doing the treatment… more than I expected… it was the only solution available.”
- U10: “They told me I would go to the hyperbaric chamber to recover more quickly… it has been very, very worthwhile… it went very well, and I’m very happy.”
- U11: “It was totally worth it.”

These accounts reflect a shared perception that HBOT contributed meaningfully to participants’ recovery, symptom improvement, or management of their underlying condition, reinforcing the value of the therapy despite the demanding and prolonged nature of the treatment.

### 3.2. Theme 2: Lived Experiences Through Interpersonal Relations with Healthcare Professionals

Participants described their interactions with the nurses who accompanied them inside the hyperbaric chamber, as well as with the hyperbaric physician. Their accounts focused primarily on the personal and professional care received from these healthcare providers.

#### 3.2.1. Hyperbaric Nurses

Participants consistently identified nurses as the professionals present with them inside the chamber. According to their descriptions, nurses played a central role in guiding, informing, and accompanying them throughout the HBOT session. Nurses provided instruction on ear pressure compensation techniques, addressed questions or concerns during treatment, and responded to any issues that arose. Participants also highlighted feeling safe because nurses continuously monitored them during the procedure.

Their experiences are illustrated in the following quotations:

- U1: “She told me, ‘Pinch your nose and blow.’”
- U2: “There was a nurse next to me who watched over you, who could see you.”
- U5: “The nurse inside the chamber started explaining things to me… I think we are very well monitored, always with the nurses—they’re there with us…”
- U6: “When we went into the chamber, there was a nurse—or a male nurse in this case—and you could see they were cheerful and attentive.”
- U7: “For new people… they would teach them how to compensate their ears.”
- U12: “I consider that they were all very, very efficient.”

These testimonies emphasize the importance of nursing care in fostering a sense of safety, emotional support, and confidence during the treatment, positioning nurses as key figures in the overall patient experience.

#### 3.2.2. Hyperbaric Physician

Participants located the hyperbaric physician in an office adjacent to the chamber and described interactions that primarily occurred before their first HBOT session. According to their accounts, the physician’s main role was to provide initial information about the treatment and its functioning. However, participants reported limited follow-up communication throughout the treatment course. They noted that although they often saw the physician in the corridor or near the chamber, they rarely interacted with him unless they specifically requested it.

Their experiences are illustrated in the following statements:

- U2: “He informed me on the very day I started… not at the end. He informed me at the beginning and in between, because—rather than formal interviews—he would come in and out a bit when we entered or left… many times we would see each other in the corridor… always outside, in the office right next to the chamber.”
- U3: “I trust the doctors.”
- U4: “He informed me and explained everything… yes, he explained it very well.”
- U5: “Before entering the chamber, he spoke with me… he explained everything… he told me that with this treatment the wound would close.”
- U7: “He attended to me very well; he asked me questions and gathered several details… he explained everything quite well.”
- U12: “Because of that, I considered all of them to be very, very effective.”

These accounts highlight the physician’s role in providing essential initial information and clinical guidance, while also revealing gaps in ongoing communication during treatment. Participants expressed trust in the physician’s expertise but noted that routine interaction was minimal unless clinically required.

#### 3.2.3. Quality of Care and Perceived Professional Support

All participants expressed high satisfaction with the care received from the hyperbaric unit staff—including nurses, chamber technicians, and the hyperbaric physician. They consistently described the staff as attentive, respectful, and supportive. Participants emphasized that nursing staff, in particular, provided continuous accompaniment throughout the treatment process, contributing to a sense of reassurance and safety.

Their perceptions are reflected in the following accounts:

- U2: “I am very satisfied with the hyperbaric chamber and with the care received inside the chamber—from the nurses, from the people who are inside helping you put on the mask, checking everything… I have nothing negative to say, only very nice things… and the rest of the staff as well.”
- U6: “…I am very happy, let that be clear. I am very satisfied with the service there, with the nurses who came and accompanied us.”

### 3.3. Theme 3: Communication Between Professionals and Participants

Participants described the type and quality of information they received from healthcare professionals, as well as aspects they believed could be improved.

#### 3.3.1. Information Received

Participants explained that the first information they received about undergoing HBOT typically came from the specialist managing their underlying condition—such as oncologists, trauma surgeons, or vascular specialists—who considered HBOT an appropriate adjunct therapy. Subsequently, participants met with the hyperbaric physician responsible for explaining the treatment process.

Their accounts reflect a range of initial explanations:

- U1: “I had had a stroke that affected my eye, and they told me that it had to be resolved, and the best way to prevent further issues for the moment was to put me in the chamber.”
- U2: “I started HBOT because it offered a solution for my condition (urological problem).”
- U3: “It was to heal a wound… I would have liked them to inform me better about everything, but it all happened very quickly. No, they didn’t inform me very well, to be honest.”
- U4: “I was pleased because it seemed like it would be a good treatment option.”
- U6: “I had already tried so many things for this pyoderma and had done so many treatments… this was another option, and they had told me that the chamber treatment might help.”
- U8: “The first time they mentioned hyperbaric therapy was two or three days after I was admitted following the accident, but because I was COVID-positive I couldn’t start. Later, after surgery and when they removed the cast and did an arthroscopy, since my cartilage wasn’t recovering, they recommended HBOT sessions.”

In some cases, information was directed primarily to family members rather than to the patient. Participants noted that this could result in gaps in their own understanding of the treatment.

- U1: “Since I went with my eldest son, he came in because many times I don’t understand or I forget… from what I saw, the doctor—a woman—was explaining everything to him first about what needed to be done…”
- U9: “I always went with my daughter, and at that moment I found everything correct.”

These accounts highlight variability in communication practices, with some participants feeling well informed while others noted insufficient or indirect communication, underscoring the importance of ensuring that information is consistently addressed to the patient.

#### 3.3.2. Aspects for Improvement in Communication

Participants identified several areas where communication could be improved. A recurrent theme was the lack of sufficient information and time dedicated to explaining the HBOT process. In some cases, professionals did not clearly identify themselves or their professional role, leaving participants uncertain about whether they were interacting with a physician, nurse, or technician—and sometimes not even knowing the person’s name.

Several participants voiced these concerns:

- U1: “I would have appreciated more time and attention.”
- U3: “A bit more information would be good, but I suppose you have to be the one to ask, right? I missed information about how it works or what the contraindications are. A bit more information would have been helpful.”
- U5: “They would need more time… If there were more time, they could prepare you outside.”
- U6: “For me personally it didn’t matter much, but I’m sure that for someone else… yes, they would have appreciated receiving the explanation outside.”
- U6: “They didn’t tell me who they were, and I didn’t ask…”
- U7: “There are always things that can be explained in different ways, but I think it would have been good to receive the correct information a few days earlier.”
- U8: “Maybe I would have liked them to tell me that it was such a small space, more information about what the chamber is like, how it works… whether I could stand up… that there is noise and temperature changes.”

Participants frequently noted gaps in information about chamber functioning, potential risks, and the need for ear pressure compensation, identifying these as priority areas for improvement.

- U2: “I wasn’t given an explanation of the chamber’s function or how it works.”
- U5: “I don’t know how the chamber works.”
- U6: “I don’t know how it works; I only know there is oxygen coming in.”
- U10: “I saw that they closed the chamber and told me to put on the mask, and when the time was up, we left… and the nurse was there to answer any question we had.”

Information transfer appeared especially challenging for older adults or those in physically or emotionally fragile situations, who sometimes struggled to understand or retain instructions:

- U8: “You have to compensate your ears… older people weren’t really told… there were people who didn’t know how to do it.”
- U10: “I don’t remember them telling me it was 14 metres… I wasn’t very focused at the time.”

Most participants stated they had not received information about the possible risks associated with HBOT. A few reported hearing informal or partial comments, but systematic risk communication was not perceived.

- U2: “I don’t know the risks of doing the treatment.”
- U5: “I know some risks, like you can’t go in with a cold—you have to tell them because you can’t compensate well.”
- U6: “I know it could explode… they always checked that we didn’t bring in lighters, but they didn’t explain it—I deduced it.”
- U10: “No one explained the risks to me.”
- U4: “Well, the doctor mentioned hearing problems or nervous crises, things like that… as far as I know, nothing more.”

Regarding knowledge of the Safety Protocol, only one participant reported knowing what to do in case of emergency; all others stated they had not received such information.

- U7: “No, I have no idea that there is any safety protocol.”
- U8: “Yes, I know what could happen and how to act in an emergency.”

Overall, these findings indicate important areas for strengthening communication, particularly regarding the explanation of risks, chamber functioning, safety procedures, and clear identification of professionals.

### 3.4. Healthcare Structures and Material Resources

This section includes three main categories: comfort inside the hyperbaric chamber (masks, seating, temperature, music, leisure activities), session scheduling, and the logistical circuit involved in patient transport to the hospital.

#### 3.4.1. Comfort Inside the Chamber

Overall, participants did not report major discomfort associated with wearing the mask, although some mentioned occasional itching, pressure from the elastic bands, or brief sensations of restricted breathing. A commonly mentioned inconvenience was the inability to speak with other participants once the mask was on.

- U2: “The mask and the tubes limit you; you can’t stand up.”
- U4: “Sometimes you had to move the mask a bit, and it would itch here.”
- U6: “If anything bothered me, it was the mask—the elastic at the back of the neck… it bothered me a bit, though it was already well adjusted.”
- U8: “Yes, sometimes I felt like I couldn’t breathe well, and I would press it a bit, but otherwise it was fine…”
- U9: “If my nose itched, I scratched it, of course… sometimes I had to move the mask a little.”

Participants described the chamber seats as comfortable, though the limited space made it difficult for taller individuals to stretch their legs. Some needed to coordinate with the person sitting opposite them to find a mutually comfortable position.

- U2: “The seat is comfortable, but the space is small… to stretch your legs you have to coordinate a bit with the person in front, so they leave you some room.”

Participants indicated that temperature inside the chamber was generally well regulated and comfortable. Some experienced mild fluctuations but did not consider them a significant source of discomfort.

- U11: “It’s curious because at the beginning I felt cold, but then not. On the first days, at the start I felt a bit warm—nothing much—and toward the end I felt a certain chill, but afterwards everything was perfectly fine.”

Music can be played during the session, but it is selected by the technician and controlled from outside the chamber. As a result, the choice is not always to everyone’s liking.

- U7: “We listened to music, but they put on Los 40 Principales… I like listening to music a lot, but I prefer religious music.”

Participants explained that they usually spoke with each other before and after the session but could not talk inside the chamber because of the mask. During treatment, they typically spent the time resting, reading, or even sleeping, describing the environment as calm and peaceful.

- U2: “Once you’re inside, since you can’t talk because of the mask—well, you can’t talk… but before reaching the pressure level, when there are fifteen or twenty minutes, you chat. Of course, the first days are different.”
- U4: “It was like a party—we had a good time. We would spend the time reading; we read a lot… there was a strong sense of peace there.”
- U5: “And besides, we had a great time, haha, because we could all talk to each other…”
- U6: “I read newspapers, magazines… a motorcycle magazine, another magazine… I always brought something.”

#### 3.4.2. Session Scheduling and Transportation to the Treatment Centre

Participants described frustration when HBOT sessions were cancelled or postponed due to factors unrelated to their health—such as local holidays, chamber maintenance, or organizational issues. These interruptions generated discomfort, as participants perceived a loss of therapeutic continuity and feared a reduction in the accumulated benefits of HBOT. Delays in the rescheduling of sessions were seen as especially problematic.

- U2: “The problem I had this time was that the treatment was interrupted because of the local festival in Palamós, and during the break they also inspected the chamber… it meant ten days without treatment.”

Transportation to the hospital was carried out either by private car or ambulance. Participants who travelled by ambulance frequently reported long waiting times, delays related to multi-patient routes, and lengthy journeys involving multiple stops before reaching the hospital. When combined with the duration of the HBOT session itself, these logistical demands resulted in participants dedicating many hours of their day to treatment. Feelings of tiredness and discomfort were commonly reported.

- U1: “If there were more… more direct services, it would be better. But they have to come here to Corçà, then go down to Palafrugell to pick someone up… and sometimes, like one day, we arrived at the clinic and were about to go in when they got a call saying they had to pick up someone else—they were rushing to leave quickly to have enough time…”
- U4: “I go to the hospital by car, by car…”
- U7: “…I left my house at 10:30 and didn’t get home until 18:30 every day; it’s very tiring…”
- U8: “Yes, because it was two hours… we had to be there at 11, and some days we started a bit earlier… it depended on the first group. Many days I felt like… I’ve lost the whole morning; those three hours felt really long…”
- U10: “The ambulance driver made everyone waste time—everyone was sitting inside waiting for him because he was at the bar.”
- U10: “Sometimes we started at 9:20, another day at 9:30, another day at 10:15… I complained twice because I said, ‘Well, it’s not my problem if the ambulance comes at 10:15—the ambulance should leave on time, and if not, it should wait for the next round. It’s not fair that those of us waiting inside have to…’”

These accounts highlight the significant logistical burden associated with HBOT for many participants, particularly those dependent on shared ambulance services. Transportation challenges influenced fatigue, daily routines, and overall treatment experience, underscoring the importance of optimizing scheduling and transport pathways.

## 4. Discussion

This phenomenological study provides an in-depth understanding of the lived experiences and needs of individuals undergoing scheduled treatment in a hospital-based multiplace hyperbaric chamber. The findings reveal that while participants perceived hyperbaric oxygen therapy as a beneficial and often essential component of their clinical management, the treatment process also generated a series of biopsychosocial demands that shaped their overall experience.

Consistent with previous qualitative research on hyperbaric therapy, participants’ initial reactions were marked by uncertainty, curiosity, or mild anticipatory anxiety linked to their limited knowledge of the chamber environment and its technical complexity [4,11,18]. Similar to the findings of Velure et al. [12] and Alilyyani et al. [10] patients describe HBOT as “entering an unknown world”, patients reported notable apprehension during the first session, particularly regarding pressure compensation and the confined space. However, these feelings diminished progressively as individuals gained experience and received guidance from nursing staff, highlighting the importance of familiarity and professional support in facilitating adaptation to treatment, an evolution also observed in the studies by Alilyyani et al. [10] MacInnes et al. [4], and Machado et al. [18].

A distinctive contribution of this study is the detailed description of physical discomforts and practical challenges experienced during treatment, some of which have also been documented in the literature by Zhang et al. [9]. While most participants managed ear pressure compensation effectively after receiving initial instruction, a minority reported intense pain or significant difficulties during the first session, requiring targeted support and close monitoring. Concerns related to elimination needs inside the chamber emerged as a major source of pre-session stress, an aspect rarely addressed in existing literature but highly relevant for patient preparation and emotional comfort. Similarly, visual changes—although transient or medically explained—generated uncertainty for some individuals, highlighting the need for clearer anticipatory guidance.

The findings also emphasize the centrality of the underlying disease in shaping patients’ perceptions of HBOT. For many, hyperbaric therapy represented a final therapeutic option or a hopeful alternative after prolonged clinical suffering. As a result, participants often experienced the chamber environment as secondary to their primary health concerns and viewed treatment as worthwhile despite associated discomforts. This aligns with previous research suggesting that perceived therapeutic benefit modulates tolerance of technical and environmental challenges during HBOT [10,18].

Interpersonal relationships, particularly with the nursing staff, played a crucial role in creating a sense of safety and continuity. Participants valued nurses’ constant presence inside the chamber, their proactive support in pressure compensation, and their capacity to generate a calm and reassuring environment. This finding reinforces the substantial contribution of nursing care to patient confidence, emotional regulation, and adaptation throughout the treatment process. In contrast, interactions with the hyperbaric physician were described as limited to initial explanations or occasional monitoring, pointing to potential opportunities for more structured follow-up to strengthen informational continuity.

Communication emerged as a critical area for improvement. Participants consistently reported gaps in the information received about chamber functioning, technical aspects, potential risks, and safety procedures. Some individuals indicated that key information was provided primarily to family members rather than directly to them, which contributed to uncertainty and reduced sense of control. These findings align with previous studies demonstrating that insufficient preparation increases pre-session anxiety and affects the global experience of HBOT [10,18,19]. Tailored communication strategies may therefore be needed to address the specific needs of older adults or those experiencing cognitive or emotional vulnerability.

Environmental and organizational factors also shaped participants’ perceptions of the treatment. Although overall comfort inside the chamber was acceptable, individuals described limitations related to restricted space, mask discomfort, and temperature fluctuations, consistent with the findings reported by Chalmers et al. [11] and Machado et al. [18]. The monotony associated with long or repeated sessions was often mitigated by resting, reading, or talking before pressurization, although mask use substantially reduced interaction once treatment had begun. Outside the chamber, logistical challenges—such as prolonged transportation times in shared ambulance services, extended waiting periods, and unexpected cancellations—generated fatigue and frustration, particularly among those undergoing lengthy treatment regimens. Interestingly, patients in the study MacInnes et al. [4] described these same organizational issues as only a ‘minor inconvenience’, suggesting variability in how such factors are perceived across different clinical contexts.

Taken together, the findings highlight HBOT as an emotionally and physically demanding process that is nevertheless perceived as highly beneficial. They underscore the need for patient-centred communication, supportive nursing care, and organizational improvements to optimize treatment experiences. By deepening our understanding of the psychosocial dimensions of HBOT, this study contributes to enhancing the quality of care in hyperbaric medicine and supports the development of strategies aligned with COREQ principles of transparency, reflexivity, and contextualized interpretation [15].

### 4.1. Limitations

This study presents several limitations that should be considered when interpreting its findings. First, the sample was composed of twelve participants from a single hospital-based multiplace hyperbaric chamber unit, which may limit the transferability of the results to other settings with different organizational structures, patient populations, or treatment protocols. Although data saturation was achieved, the experiences reported may not fully represent the diversity of perceptions among all individuals undergoing hyperbaric oxygen therapy.

Second, participants were recruited consecutively among those who had completed at least ten treatment sessions, which may have introduced selection bias. Individuals who discontinued treatment early or who had particularly negative experiences may not be represented. Additionally, although interviews were conducted within a defined time frame after HBOT sessions, subjective recall may still have been influenced by the time elapsed since treatment, particularly for participants interviewed outside the clinical setting. This potential recall bias was considered during data analysis and interpretation.

Finally, interviews were conducted in diverse environments, participants’ homes, a health centre, or the hyperbaric medicine unit, based on the participants’ preferences. Although this flexibility supported comfort, it may have introduced variability in the interview context and influenced how participants expressed their experiences.

### 4.2. Recommendations for Further Research

Future research should further examine patients’ informational needs before and during hyperbaric oxygen therapy, particularly regarding chamber functioning, safety procedures, and pressure compensation techniques. Studies exploring best strategies for delivering clear, tailored, and accessible information—especially for older adults or individuals in a vulnerable physical or emotional state—may contribute to improving patient preparedness and reducing pre-treatment uncertainty.

Additional qualitative research involving larger and more diverse populations could deepen our understanding of the psychosocial experiences identified in this study, including anxiety related to the unfamiliar environment, concerns about elimination needs, and the impact of long transportation times. Comparative studies across different hyperbaric units may also help identify organizational factors that influence patient comfort, satisfaction, and adherence to treatment.

Given the central role of nursing staff in creating a sense of safety and support inside the chamber, future work should explore nurse–patient communication dynamics and the effectiveness of specific nursing interventions aimed at enhancing comfort, reducing anxiety, and improving patients’ ability to manage treatment-related challenges.

Finally, mixed-methods or longitudinal designs could provide additional insight into how perceptions and needs evolve across multiple sessions and how these experiences relate to therapeutic outcomes. Such evidence may support the development of patient-centred educational programmes and organizational improvements in hyperbaric medicine services.

### 4.3. Implications for Practice

The findings of this study highlight several areas where clinical practice in hyperbaric medicine can be improved to better support patients undergoing treatment in a multiplace hyperbaric chamber. First, enhancing patient education is essential. Clear, consistent, and tailored information about chamber functioning, pressure compensation techniques, potential risks, and safety procedures should be systematically provided before the first session. Direct communication with patients, rather than relying on family members as intermediaries, may reduce uncertainty and foster a sense of control.

Nursing practice plays a central role in shaping patients’ experiences inside the chamber. In this study, nursing staff refers specifically to registered nurses with direct responsibility for patient monitoring, safety, and emotional support inside the hyperbaric chamber, in accordance with their professional scope of practice. The supportive presence of nurses was strongly valued, underscoring the importance of maintaining close monitoring, reassurance, and proactive guidance throughout the session. Strengthening structured communication protocols and ensuring that all staff members introduce themselves and clarify their professional roles may further improve the therapeutic relationship and patient confidence.

Attention to comfort and environmental conditions inside the chamber is also important. Although generally acceptable, issues related to mask discomfort, limited space, and temperature fluctuations suggest opportunities for ergonomic adjustments and personalized comfort measures. Providing strategies to manage elimination needs before treatment may also reduce patient distress. From a patient-centred perspective, offering the possibility to choose music during treatment—introduced at the time of informed consent and treatment explanation—may enhance comfort, reduce anticipatory stress, and support treatment adherence.

At an organizational level, optimizing scheduling processes and reducing unnecessary interruptions or cancellations could prevent treatment delays and minimize the loss of clinical benefit. Participants described cancellations or postponements due to administrative factors as particularly distressing, highlighting the importance of proactive communication and careful coordination. For patients dependent on ambulance transport, reviewing transport logistics and reducing waiting times may significantly improve their overall experience and reduce fatigue associated with prolonged daily routines.

Overall, implementing patient-centred communication, improving logistical coordination, and reinforcing the supportive role of nursing staff can contribute to a more positive and efficient treatment experience in hyperbaric units.

## 5. Conclusions

This study explores the perceptions and needs of individuals undergoing scheduled treatment sessions in a multiplace hyperbaric chamber. The findings show that patients experience the therapy as a largely positive and beneficial process, particularly in relation to improvements in their underlying conditions, which were often perceived as their main concern. Initial uncertainty or mild anxiety was common before the first session, mainly due to unfamiliarity with the chamber environment, but generally decreased as patients gained experience.

Participants reported specific physical and emotional needs during treatment, including concerns related to ear pressure compensation, elimination needs, and the impact of visual changes. Despite these challenges, most felt safe and well supported by the nursing staff inside the chamber, highlighting the essential role of nurses in providing guidance, reassurance, and continuous monitoring.

Communication emerged as a key area for improvement. Participants identified gaps in the information received about the functioning of the chamber, potential risks, compensation techniques, and safety protocols. For some, information was provided primarily to family members rather than directly to them, which contributed to uncertainty and unmet informational needs.

Environmental and organizational aspects also shaped the overall experience. Comfort inside the chamber was generally acceptable, although factors such as limited space, mask discomfort, and temperature fluctuations were mentioned. Outside the chamber, issues related to scheduling, session cancellations, and long transportation times, especially for those dependent on ambulance services, created fatigue and inconvenience.

Overall, this study highlights that, while the hyperbaric chamber treatment is perceived as effective and worthwhile, patients have clear informational, emotional, and organizational needs that must be better addressed. Strengthening patient education, improving communication, and optimizing logistical processes may enhance the treatment experience and support patient-centred care in hyperbaric units.

## Figures and Tables

**Table 1:** Semi-structured interview guide.

What information would you have liked to receive when you were first told that you would need hyperbaric oxygen therapy?
How did you feel in the days prior to starting treatment? And the night before the first session?
What sensations or impressions did you experience on the first day you entered the hyperbaric chamber?
Have these sensations persisted throughout the rest of the sessions?
Do you believe that undergoing the treatment has been worthwhile considering the benefits obtained?
Have your expectations regarding the therapeutic effects of the treatment been met?
Questions exploring prior knowledge about the hyperbaric chamber: − What did you know about it before starting treatment? − From whom and when did you learn this information? − Specific topics explored: chamber functioning, potential risks, pressure-compensation mechanisms, patient role inside the chamber, and safety protocol.
Targeted questions, if not previously addressed during the interview, related to: − anxiety − pain − claustrophobia − use of time during sessions − noise − temperature − comfort − mask-related discomfort (dryness, pressure, etc.) − nteraction with other treatment users − visual acuity changes

**Table 2:** Characteristics of the study participants.

ID	Gender	Age	Number of Sessions Received	Pathology
U1	Male	78	14	Central retinal artery occlusion (CRAO)
U2	Male	83	30	Soft tissue radionecrosis (cystitis)
U3	Male	51	10	Open fractures with crush injury
U4	Male	72	52	Soft tissue radionecrosis (cystitis, proctitis)
U5	Female	76	40	Ischaemic ulcers
U6	Male	62	73	Perineal pyoderma gangrenosum secondary to immunotherapy
U7	Male	71	54	Soft tissue radionecrosis (proctitis)
U8	Female	25	40	Closed crush injuries, tissue viability clinically judged to be at risk
U9	Female	59	20	Compromised skin grafts and musculo-cutaneous flaps
U10	Female	69	20	Crush Injury without fracture
U11	Male	84	44	Rectal bleeding following radiotherapy for prostate cancer
U12	Male	71	30	Martorell ulcers in the lower limbs

## Data Availability

The data presented in this study are available on request from the corresponding author due to privacy and ethical restrictions.

## Public Involvement Statement

No public involvement in any aspect of this research.

## Guidelines and Standards Statement

This manuscript was drafted against the Consolidated criteria for reporting qualitative research (COREQ) [15].
